# Supplementary material for: Computational multiphysics modeling of radioactive aerosol deposition in diverse human respiratory tract geometries
Source: Commun Eng. 2024 Nov 1;3:152. doi: 10.1038/s44172-024-00296-z (PMC11530636; doi:10.1038/s44172-024-00296-z)
Supplement: Supplementary file 2 — Supplementary Information [file 44172_2024_296_MOESM2_ESM.pdf]

Supplementary Information

Ignacio R. Bartol[1], Martin S. Graffigna Palomba[1], Mauricio E. Tano Retamales[2,*], and Shaheen A. Dewji[1,*]

[1]Georgia Institute of Technology, 1770 State St NW, Atlanta, GA 30332, USA
[2]Idaho National Laboratory, 955 MK Simpson Boulevard, Idaho Falls, ID 83415, USA
[*]Corresponding Author: shaheen.dewji@gatech.edu; Co-Corresponding Author: mauricio.tanoretamales@inl.gov

ABSTRACT

This is the supplementary information material for the manuscript titled "Computational Multiphysics Modeling of Radioactive Aerosol Deposition in Diverse Human Respiratory Tract Geometries"

Supplementary Notes 1

Verification and Validation

An exhaustive verification and validation effort was performed to rigorously validate the accuracy of our CFD simulations generated through our hybrid automated workflow adhering to the protocols established by the National Program for Applications-Oriented Research in CFD (NPARC) Alliance Verification and Validation guidelines[1]. For this study, one geometry that features seven generation of bronchi was selected from our database. This geometry was utilized to generate seven distinct mesh configurations featuring varying degrees of refinement, as graphically depicted in Suppplementary Figure 1. These meshes were subsequently used to solve steady-state conditions under a constant inlet airflow rate of $Q = 90$ L min^{-1} from the main article, with except of using SIMPLE algorithm instead of PIMPLE algorithm, since it is a steady state simulation, the specific solver used was `simpleFoam` in OpenFOAM.

Mesh generation was executed using SnappyHexMesh, as will be further explained in the Supplementary Methods section, a meshing utility within the OpenFOAM software suite[2]. Element base sizes spanned a range from 1.84 mm for the coarsest mesh to 0.087 mm for the most refined mesh. Five intermediate meshes were created to augment our dataset, resulting in an ensemble of seven distinct computational domains. Refinement ratios (r) between adjacent refinement levels were consistently maintained within the 1.25 to 2 range, according to NPARC, and to maintain the mesh quality.

When generating the meshes, all the parameter values in SnappyHexMesh were carefully adjusted to pass quality mesh checks. All the meshes were tested under astringent quality checks that accounted for Overall domain bounding box, Mesh geometric directions, Mesh solution directions, Boundary openness, Max cell openness, Max aspect ratio, Face area magnitudes, Cell volumes, Mesh non-orthogonality, Face pyramids, Skewness, Coupled point location match, Edge length, Faces with concave angles, Face flatness, Faces with a ratio between projected and actual area < 0.8, Cell determinant (well-posedness), Concave cells (using face planes), Face interpolation weight, Face volume ratio. To report the cell quality, in Suppplementary Figure 1, on the bottom row, the distribution of the skew value for each cell can be seen in the form of a histogram. The skew value should be as close as possible to zero, and the average skew value is always well below the < 0.3 limit.

An additional high-fidelity simulation was conducted using a mesh featuring an even finer element base size of 0.054 mm to establish a benchmark for convergence analysis. This simulation serves as our 'exact solution' for subsequent analyses.

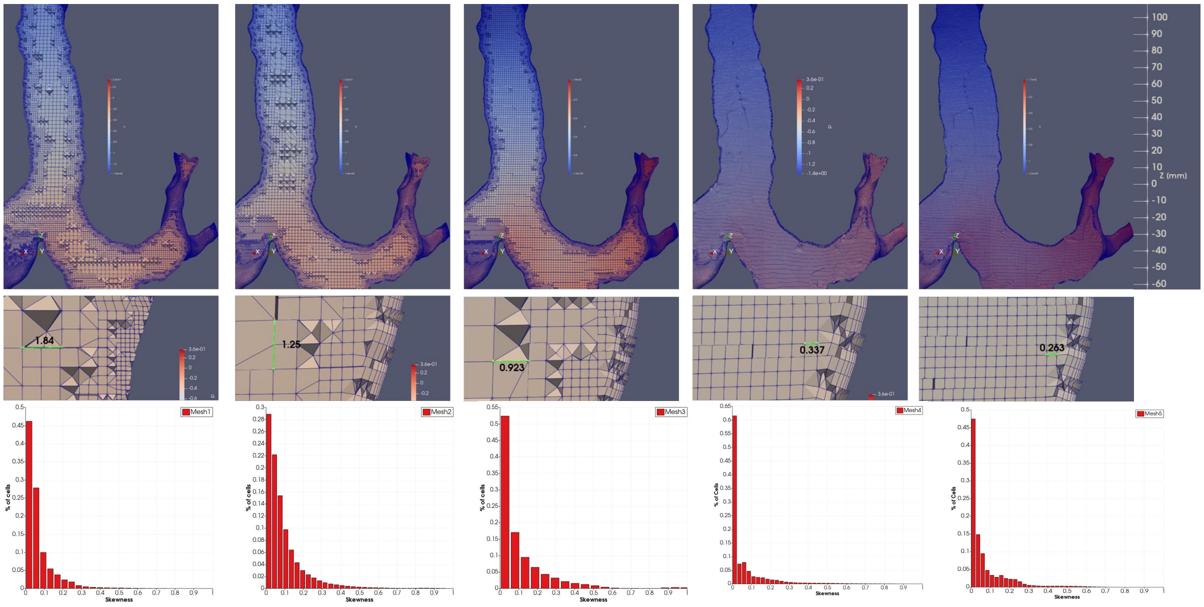

Supplementary Figure 1. Mesh quality analysis for the meshes used in the mesh independence test. A subset view of five of the seven distinct meshes employed in the mesh independence analysis is presented. The upper row depicts a broad overview of the geometric configurations, while the middle row provides a magnified view of each mesh. The distance (in millimeters) denoted corresponds to the edge length of the largest cubic hexahedron within the mesh. The two remaining mesh images are excluded for visual presentation and are consistent with the characteristics depicted in the five depicted cases. The bottom row shows the skew value for each mesh in a histogram fashion; the closer the skew to zero, the better the mesh. All the meshes scored an average skewness of less than 0.3, demonstrating the meshes' quality.

Residuals of each simulation were confirmed to converge within $< 10^{-4}$. Three distinct metrics were further employed to

quantify the quality of the convergence behavior: Order of Grid Convergence; Grid Convergence Index (GCI); and Asymptotic range of convergence. The Order of Grid Convergence was first calculated employing a least-squares linear fit, as described by Equation 1, where h represents the largest side of the largest hexahedron generated by the meshing tool, E is the error between the exact and mesh-based solutions, p_c is the order of convergence of the employed method, and C is an arbitrary constant. Specifically, p_c was determined for both the average pressure and the average turbulent kinetic energy (k) using an arbitrary cross-sectional slice within the trachea in the geometry. The resultant $p_c(E(p)) = 1.61 \pm 0.30$ and $p_c(E(k)) = 1.66 \pm 0.17$ for pressure and turbulent kinetic energy, respectively, were observed to be slightly lower than their theoretical counterparts; this is attributable to the imposed boundary conditions, numerical models, and mesh configurations. Suppplementary Figure 2 displays the linear fit results and the data points of the mesh convergence studies.

$$\log(E) = \log(C) + p_c \ \log(h) \tag{1}$$

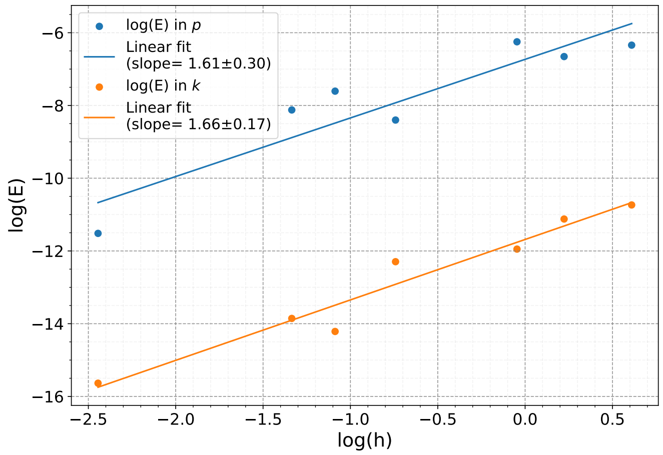

Supplementary Figure 2. Grid convergence order analysis. Observed grid convergence order (p_c) across seven distinct lower respiratory tract meshes, evaluated based on errors in pressure (p) and turbulent kinetic energy (k). The mesh spacing (h) is defined as the length of the largest edge of the maximum hexahedral volume generated using the SnappyHexMesh tool. Generally, h corresponds to the pre-selected background hexahedral base element size.

Following the methodology outlined in the study by Roache et al.[3], the GCI was calculated for three selected meshes to evaluate discretization errors and ascertain grid-independent CFPD simulation results. Initially, three distinct grids with a consistent grid refinement ratio r across two combinations (fine-to-medium and medium-to-coarse) were selected, as defined by Equation 2. To mitigate computational load, the coarsest meshes meeting this criterion were prioritized.

The designated grid spacings were defined as: $h_{\text{fine}} = 0.48\,\text{mm}$, $h_{\text{medium}} = 0.95\,\text{mm}$, and $h_{\text{coarse}} = 1.84\,\text{mm}$. The refinement ratios were calculated to be $r_{\text{mf}} = 1.98$ for medium-to-fine and $r_{\text{cm}} = 1.94$ for coarse-to-medium. The minor deviation between these ratios arises from OpenFOAM's background mesh generation process. Specifically, when utilizing the BlockMeshDict tool, the user defines a box encapsulating the domain and subdivides it into cubic-like elements. During refinement, the domain is divided an integer number of times to approximate half of the original block sizes. This refinement leads to minor variances in refinement ratios for the two grid combinations. This rigorous approach ensures a robust evaluation of the CFPD simulations' sensitivity to grid refinement, thereby contributing to the validity of our findings.

The GCI was then computed using equations 3 and 4, using a safety factor of $F_s = 1.25$ and ϕ is the solution variable. The average pressure p and average turbulent kinetic energy k in an arbitrary axial slice within the trachea was used as the ϕ solution variables. The results for the GCI and the asymptotic range of convergence can be seen in Supplementary Table 1, where it can be seen from the asymptotic range of convergence is close to one and indicates that the solutions are within the asymptotic range of convergence. The error in the pressure will be less than 0.46% and in the turbulent kinetic energy less than 3.22%. The details for each mesh used in the convergence studies are given in Supplementary Table 2. The faces per cell in Supplementary Table 2 refer to the average number of faces per cell present in each mesh, which relates to the quality of the hexahedron-dominant mesh generated by SnappyHexMesh. The closer the value to 6, the fewer elements with more than six faces were introduced in the meshing process. Elements with multiple faces can lead to local instabilities if they are significant in the mesh, but in the worst case, 8% of the elements were not hexahedrons. In the best scenario, 99% of the elements were hexahedrons.

$$r = \frac{h_{\text{medium}}}{h_{\text{fine}}} = \frac{h_{\text{coarse}}}{h_{\text{medium}}} \simeq 2 \qquad (2) \qquad \text{GCI}_{\text{fine}} = \frac{F_s}{r^p - 1}\left|\frac{\phi_{\text{fine}} - \phi_{\text{medium}}}{\phi_{\text{fine}}}\right| \qquad (3) \qquad \text{GCI}_{\text{coarse}} = \frac{F_s\, r^p}{r^p - 1}\left|\frac{\phi_{\text{medium}} - \phi_{\text{coarse}}}{\phi_{\text{medium}}}\right| \qquad (4)$$

ϕ	Pressure p	Turbulent K.E. (k)
$\text{GCI}_{\text{coarse}}$	1.47%	9.51%
GCI_{fine}	0.46%	3.22%
Asymptotic range of convergence	1.05	0.97

Supplementary Table 1. GCI index for the medium-fine and coarse-medium meshes. The asymptotic range of convergence is approximately one for both fields indicating the solutions are converged.

**Geometry number**	**Element** base size	**Number of elements**	**Faces per cell**
1	1.84 mm	2,077,219	6.49
2	1.25 mm	2,628,353	6.46
3	0.95 mm	2,994,346	6.05
5	0.47 mm	3,926,570	6.05
6	0.32 mm	4,501,284	6.25
4	0.19 mm	5,371,045	6.22
7	0.087 mm	7,607,261	6.10

Supplementary Table 2. Details of the seven meshes used for for the mesh convergence studies. The table is order in decreasing size of the base hexahedral mesh size used and increasing size number of elements in the final mesh.

Airflow structures and particle deposition

Suppplementary Figure 3 present the main flow velocity and secondary velocity profiles at various cross-sections for lower HRTs, for people representative of a population with an average trachea diameter from our analysis.

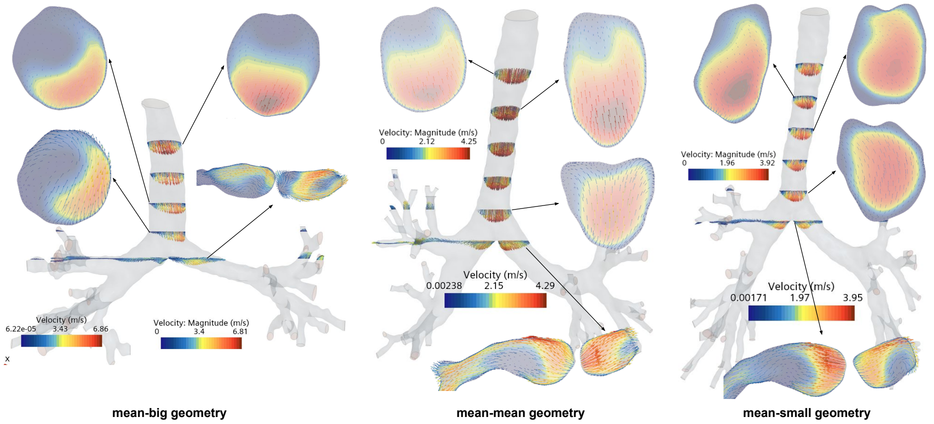

Supplementary Figure 3. Flow velocity profiles and secondary velocity flow in the different parts of the trachea and the main bronchi for a set of people from the clustering process. The snapshot corresponds to the inhalation peak time step (i.e. 0.5 seconds in physical time). In particular, those geometries belong to HRT geometries who lied on the mean trachea diameter with a big (mean-big geometry), mean (mean-mean geometry) and small (mean-small geometry) carina angle clusters respectively.

To further elucidate complex flow structures in the upper respiratory tract, Suppplementary Figure 4 displays streamlines at the peak of inhalation (i.e., at 0.5 s into the simulation), with turbulent viscosity ratio represented as the scalar field.

For this study the turbulent viscosity ratio was used as a figure of merit to identify where turbulence could be occurring. Previous studies have also identified a laryngeal jet in this region causing turbulence to be propagated downstream[45]. Moreover, Suppplementary Figure 4 demonstrates the mechanism of swirling flow entering the trachea, as well the recirculating flow observed in the glottis close to the oral cavity for nasal inhalation breathing condition.

The pressure drop was analyzed at the peak of the inhalation for a flow with particles and for a flow without particles to analyze the influence of the particles in the airflow field. The summation of the pressure drop from the inlet to all the outlets for all the geometries analyzed is showcased in Supplementary Table 3. One pressure drop is calculated only based on the airflow ΔP_{flow}, and the second pressure drop is calculated with particles $\Delta P_{\text{particles}}$, the relative difference is $\varepsilon(\Delta P)$.

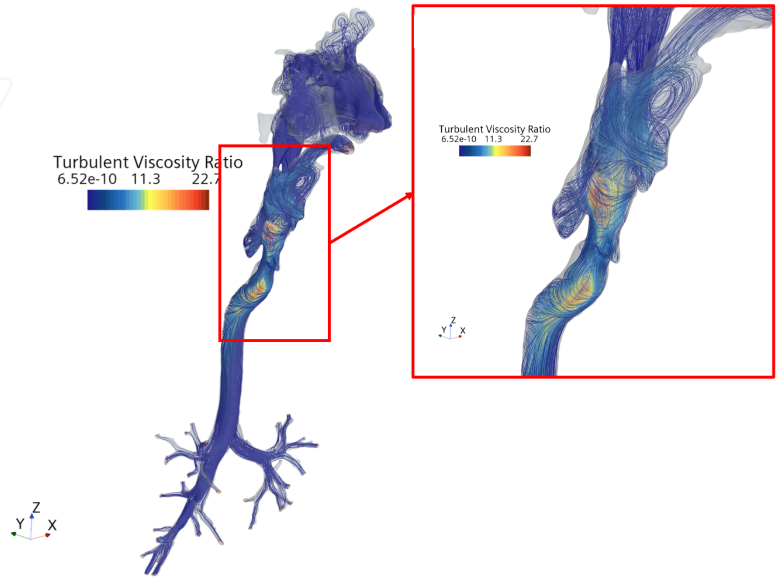

Supplementary Figure 4. Streamlines displaying the turbulent viscosity ratio as the scalar fiend at the peak of the inhalation. Indication of turbulent flow can be seen formed in the larynx region and the trachea constriction, where a detailed view can be seen on the right hand side of the picture.

Geometry	ΔP_{flow}	$\Delta P_{\text{particles}}$	$\varepsilon(\Delta P)$
mb	1.19×10^1	1.33×10^1	11%
mm	1.29×10^1	1.14×10^1	-14%
ms	1.08×10^1	1.03×10^1	-5%
bb	1.30×10^1	1.22×10^1	-6%
bm	8.66×10^0	1.10×10^1	21%
bs	4.27×10^0	6.61×10^0	35%
sb	1.35×10^1	1.21×10^1	-12%
sm	1.21×10^2	1.04×10^2	-16%

Supplementary Table 3. Summation of the pressure drop from the inlet to all the outlets in the geometry, having only the airflow ΔP_{flow} and the airflow plus particles $\Delta P_{\text{particles}}$. The relative difference is shown as $\varepsilon(\Delta P)$.

PHITS simulation

Supplementary Table 4 presents the absorbed dose normalized per unit source derived from the point source distribution generated by the CFPD simulation.

Organ	Volume $[cm^3]$	Dose $[pGy$/source$]$	Relative error [%]
Right Lung	1342.1	0.609	0.072
Left Lung	1123.8	1.069	0.062
Liver	1800.9	0.0074	1.10
Stomach	151.5	0.32	1.05
Bladder	39.7	2.57×10^{-3}	11.40
Small Intestine	672.9	0.0049	1.62
Ascending Colon	93.5	0.0025	6.12
Descending Colon	93.5	0.0075	4.41
Sigmoid Colon	41.6	5.59×10^{-3}	14.0
Transverse Colon	124.7	0.11	3.06

Supplementary Table 4. Dose per unit source to the most important organs surrounding the lungs in the human body, using an ^{131}I as the radionuclide for the point sources.

Supplementary Methods

Geometry Reconstruction

The CT scans acquired from various databases were central to our methodology, with a specific focus on reconstructing the 3D geometry of the human respiratory tract (HRT). The average voxel dimensions and its statistics are given in Supplementary Table 5.

For the upper respiratory tract, which includes structures such as the trachea, pharynx, sinuses, oral cavity, and nasal cavity an updated version of the algorithm by Cercos-Pita J.L. et al.[6] was implemented. The algorithm was enhanced to accommodate scans with a field-of-view (FOV) exceeding the dimensions of the human cranium, a feature missing in the original algorithm. Additionally, it was modified to capture multiple anatomical structures, such as the nasal and oral cavities, while discarding disconnected or small-volume bodies. The Pydicom framework was updated for this task, and deprecated VTK-GDCM dependencies were replaced with either VTK or GDCM.

Further refinements to the algorithm allowed the segmentation of the oral cavity when connected to the pharynx. The revised segmentation function permits the inclusion of not only the nostrils but also the oral cavity and sinuses. A criterion for identifying the oral cavity based on volume ranges of $(3-9.66)\text{cm}^3$ as reported by Rana SS. et al.[7] was added. The lower limit for the oral cavity volume was determined through iterative trials, and the upper limit represents the mean volume plus one standard deviation derived from empirical data.

In parallel, the lower respiratory tract, extending from the trachea to the 5th to 7th generation of bronchi, was analyzed using a different set of CT scans. A 3D-UNet architecture was employed, based on the Navi-Airway framework[8]. Post-processing tools were adapted from the airway extraction methodologies detailed in the work of Garcia-Uceda[9]. This deep learning approach was trained using the EXACT'09 dataset, a benchmark in the field[10], and subsequently validated it on a separate dataset. Following the reconstruction, an automated parametrization process was implemented. This process quantified key metrics, including the length and average diameter of the trachea, the G0-to-G1 branching angle, and the overall volume of the reconstruction.

This parametrization provides a heterogeneous mix of phenotypical and non-phenotypical metrics characterizing the CT scan database for the lower HRT. Random forest regression was applied to identify the most predictive features of the lower HRT for estimating an individual's age and weight. The final preparatory step for CFD/CFPD simulations involves smoothing the 3D geometry, defining inlets/outlets, and merging the upper and lower respiratory tract geometries. Depending on the computational power available, this step may be circumvented for simulations involving the complete HRT.

Supplementary Table 5. Statistics for the voxel dimensions of the CT scans used in this work.

	x	y	z
Mean [mm]	0.67	0.67	0.95
Standard Dev. [mm]	0.07	0.07	0.17
Min [mm]	0.55	0.55	0.60
Max [mm]	0.78	0.78	1.25

Geometry Pre-Processing

A Python script implemented in Blender[11] applies a Laplacian smoothing filter to refine the geometry. This filter incorporates volume conservation and normalization, thus ensuring that the finer bronchial structures are retained and avoiding sharp wall edges that may lead to numerical inaccuracies in the simulations. The key parameters for this filter are: $\lambda_{\text{smooth}} = 1.5$, $\lambda_{\text{border}} = 2.5$, and the number of iterations $n = 10$. It is worth noting that higher values of λ_{smooth} and n can adversely affect the preservation of the original geometry, whereas λ_{border} controls the degree of bronchial shrinkage. Before smoothing, remeshing is carried out to quadruple the number of triangles in the STL file, thereby preventing geometric degradation during the smoothing operation.

Following geometry smoothing of the upper and lower HRT, the next task is to designate inlet and outlet surfaces. To date, no automated or semi-automated methodologies exist for this process, making it particularly time-consuming for complex geometries, mainly when the geometry encompasses $2^{n^{\text{th}}} + I$ bronchi generations. Here, n^{th} denotes the number of bronchi generations, and I can vary based on whether the nasal cavity, oral cavity, or both are included. In this study, bronchi generations ranged from 5th to 7th, partially or fully covering the tracheobronchial (BB) region as defined in ICRP Publication 66[12]. A Python script was developed in Blender to facilitate this operation, employing boolean operations for surface segmentation.

The semi-automated script proceeds as follows, with steps requiring manual intervention indicated in bold text:

1. **Create a disc in Blender with a diameter ranging from 5mm to 10mm and a height of approximately 1mm along the Z-axis**. Lets define the disc's normal vector as $\mathbf{n}_d$.

2. **Manually identify an outlet and select three vertices that approximate the desired cut plane**. Denote these points as $\mathbf{x}_0, \mathbf{x}_1, \mathbf{x}_2$.

3. Compute the plane's normal vector through the cross product of the selected points and determine the centroid among them: $\mathbf{n}_\mathrm{p} = (\mathbf{x}_1 - \mathbf{x}_0) \times (\mathbf{x}_2 - \mathbf{x}_0)$, $\mathbf{x}_\mathrm{p} = \frac{\mathbf{x}_0+\mathbf{x}_1+\mathbf{x}_2}{3}$.

4. Align the disc's normal vector with that of the plane: $\mathbf{n}_\mathrm{d} = \mathbf{n}_\mathrm{p}$.

5. Translate the disc so its center coincides with the centroid $\mathbf{x}_\mathrm{p}$.

6. Execute a boolean difference operation using the HRT geometry as the main body and the disc as the tool body.

7. Repeat steps 2 through 6 until all outlets have been effectively segmented.

8. Retain only the geometry with the largest volume, removing any disconnected structures generated during the operation.

Subsequently, each STL surface must be categorized as a wall, inlet, or outlet for OpenFOAM to facilitate the simulation. In the case of StarCCM+, all such surfaces should be concatenated into a single STL file containing multiple STL surfaces.

The process of matching the upper and lower HRT was challenging due to the 3D geometrical reconstruction of those parts coming from different subjects. Therefore, the criteria to reconstruct the four geometries in this work were as follows:

1. Initially, it was filtered by sex and similar weight (±10 kg). Once a match was identified, we individually examined the 3D reconstructions of the lower and upper HRT and selected the most similar tracheal diameter. We did not use age for matching since it was a restrictive criterion given that we had only 40 3D reconstructions of the HRT.

2. For potential candidate pairs, we referred back to the source CT scans to ensure that the chest CT scan region overlapped with the head-neck CT scan region. If no overlap was present, the pair was discarded to avoid missing segments in the full HRT reconstruction.

3. Using coronal cuts and anatomical reference points, we matched the upper and lower respiratory tracts from the CT scans. This step was crucial for accurately aligning the 3D reconstructions and ensuring proper integration by removing overlapping sections.

CFPD Modelling

The boundary conditions for the velocity $\mathbf{u}$ were chosen to replicate a representative respiratory cycle of a human subject under heavy-exercise breathing conditions. The average respiratory cycle with its standard deviation is represented in Suppplementary Figure 5, the data was taken from experimental results from Silverman et al.[13]. The average respiratory cycle was approximated by a sin function, using a period of $T = 2$ seconds. This shorter simulation time will help reduce the computational costs while maintaining the representation of a respiratory cycle.

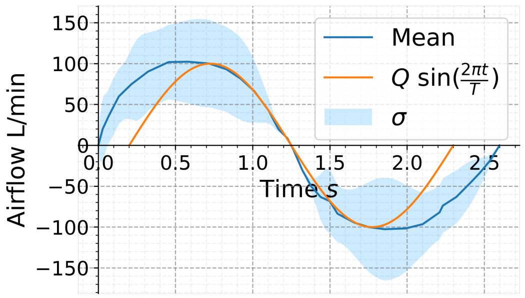

Supplementary Figure 5. Airflow for a respiratory cycle for the mean population and its standard deviation σ using Silverman et al. experimental data[13]. A negative airflow in this context represents exhalation, and a positive airflow means inhalation. An approximation to the mean respiratory cycle by a sin function is plotted in orange. The blue line represents the mean values from the original experimental data, and the shade represents the standard deviation.

For a given flowrate $Q(t) = Q_\mathrm{max} \sin\left(\frac{2\pi t}{T}\right)$, the inlet area in each geometry is calculated, and the magnitude of the inlet velocity is determined using $||\mathbf{u}|| = \frac{Q}{A}$. In OpenFOAM, this is realized using custom boundary conditions, while StarCCM+ field functions serve the same purpose.

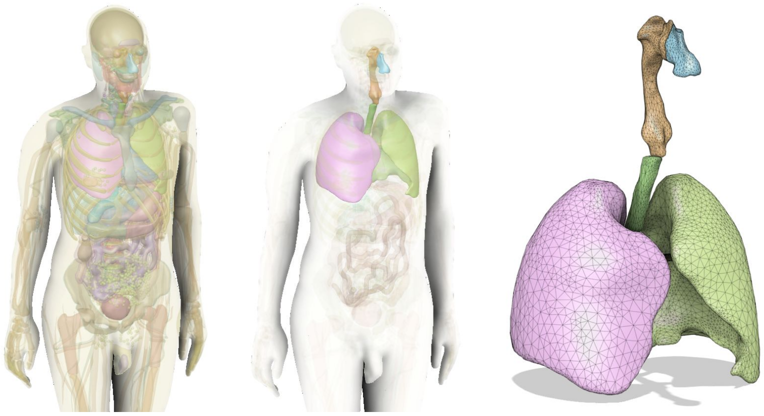

Supplementary Figure 6. Detail of the adult Mesh-type Reference Computational Phantom (MRCP). Overview of the male MRCP phantom (Left), the Human Respiratory Tract (HRT) and the lungs are highlighted (middle).The HRT and the lungs isolated from the rest of the phantom (Right).

PHITS Model Definition

In Suppplementary Figure 6 is given the general structure of the MRCP phantom and the generic HRT modeled within.

A script was done in Python to read the track files from the CFPD simulation in StarCCM+ and properly scale and rotate the particle cloud to fit the HRT in the MRCP phantom better. In Suppplementary Figure 7, the original particle distribution overlapped with the HRT of the MRCP phantom it is displayed. The tracheal carina was used as the point of reference to align the CFPD particle cloud with the MRCP phantom to match the spatial location. Afterward, using rotation operations, the particle cloud is rotated as a rigid body (i.e., the relative distance between points does not change) to match the inclination grade of the MRCP trachea.

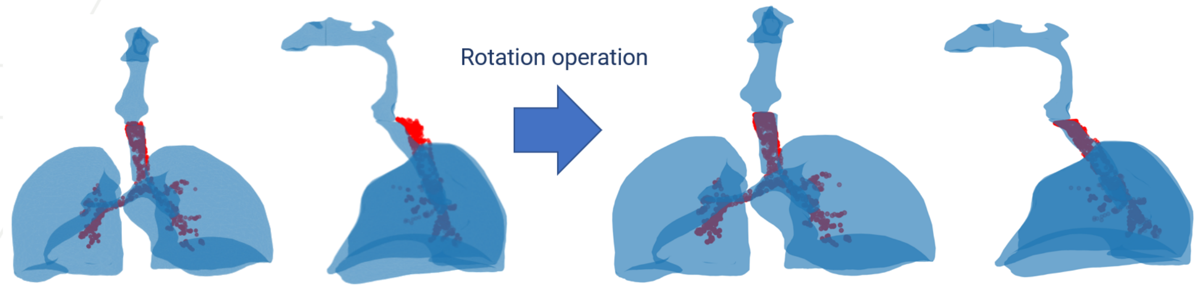

Supplementary Figure 7. Superposition of the particle deposition profiles obtained via Computational Fluid and Particle Dynamics (CFPD) simulations, on the Human Respiratory Tract (HRT) modeled in the Mesh-type Reference Computational Phantom (MRCP) used in this work..Original (left) and post-processed (right) particle distribution profiles from the CFPD simulations overlapped in the HRT and lungs male MRCP phantom geometry.

Since PHITS can not handle more than 500 point sources per simulation, the simulation is therefore divided into different batches with a maximum of 500 point sources, which means that in each run, only 250 particles can be input (each particle is two point sources, one for the γ decay and another one for the β^- decay mode). For this particular case, were 1154 particles to be simulated, resulting in five batches. The activity for each particle is assigned to be proportional to its volume, meaning $A_i = \frac{V}{a}\rho$, where a is the specific activity in [Bq kg^{-1}], ρ is the particle's density, and V is the particle's volume.

References

1. Slater, J. W., Dudek, J. C. & Tatum, K. E. The nparc alliance verification and validation archive. In *2000 Fluids Engineering Summer Conference*, no. NASA/TM-2000-209946 in ASME 2000-FED-11233 (2000).
2. Weller, H. G., Tabor, G., Jasak, H. & Fureby, C. A tensorial approach to computational continuum mechanics using object-oriented techniques. *Comput. Phys.* **12**, 620–631, DOI: 10.1063/1.168744 (1998).
3. Roache, P. J., Ghia, K. N. & White, F. M. Editorial policy statement on the control of numerical accuracy (1986).

4. Hofmann, W. Modelling inhaled particle deposition in the human lung—a review. *J. Aerosol Sci.* **42**, 693–724, DOI: 10.1016/j.jaerosci.2011.05.007 (2011).
5. Kleinstreuer, C. & Zhang, Z. Airflow and particle transport in the human respiratory system. *Annu. Rev. Fluid Mech.* **42**, 301–334, DOI: 10.1146/annurev-fluid-121108-145453 (2010).
6. Cercos-Pita, J. L., Cal, I. R., Duque, D. & de Moreta, G. S. Nasal-geom, a free upper respiratory tract 3d model reconstruction software. *Comput. Phys. Commun.* **223**, 55–68, DOI: 10.1016/j.cpc.2017.10.008 (2018).
7. Rana, S., Kharbanda, O. & Agarwal, B. Influence of tongue volume, oral cavity volume and their ratio on upper airway: A cone beam computed tomography study. *J. Oral Biol. Craniofacial Res.* **10**, 110–117 (2020).
8. Wang, A., Tam, T. C. C., Poon, H. M., Yu, K.-C. & Lee, W.-N. Naviairway: a bronchiole-sensitive deep learning-based airway segmentation pipeline for planning of navigation bronchoscopy. *arXiv preprint arXiv:2203.04294* DOI: 10.48550/arXiv.2203.04294 (2022).
9. Garcia-Uceda, A., Selvan, R., Saghir, Z., Tiddens, H. A. & de Bruijne, M. Automatic airway segmentation from computed tomography using robust and efficient 3-d convolutional neural networks. *Sci. Reports* **11**, 16001, DOI: 10.1038/s41598-021-95364-1 (2021).
10. Lo, P. *et al.* Extraction of airways from ct (exact'09). *IEEE Transactions on Med. Imaging* **31**, 2093–2107, DOI: 10.1109/TMI.2012.2209674 (2012).
11. Community, B. O. *Blender - a 3D modelling and rendering package*. Blender Foundation, Stichting Blender Foundation, Amsterdam (2018).
12. ICRP. Human respiratory tract model for radiological protection icrp publication 66. *Oxford: Pergamon* (1994).
13. Silverman, L. *et al.* Air flow measurements on human subjects with and without respiratory resistance at several work rates. *Arch. Indust. Hyg. & Occup. Med.* **3**, 461–78 (1951).
