# Supplementary material for: Computational multiphysics modeling of radioactive aerosol deposition in diverse human respiratory tract geometries
Source: Commun Eng. 2024 Nov 1;3:152. doi: 10.1038/s44172-024-00296-z (PMC11530636; doi:10.1038/s44172-024-00296-z)
Supplement: Supplementary file 3 — Reporting Summary [file 44172_2024_296_MOESM3_ESM.pdf]

nature portfolio

Corresponding author(s): DAPR COMMSENG-23-0605-B

Last updated by author(s): 9/12/2024

Reporting Summary

Nature Portfolio wishes to improve the reproducibility of the work that we publish. This form provides structure for consistency and transparency in reporting. For further information on Nature Portfolio policies, see our Editorial Policies and the Editorial Policy Checklist.

Statistics

For all statistical analyses, confirm that the following items are present in the figure legend, table legend, main text, or Methods section.

n/a	Confirmed	
☐	☒	The exact sample size (*n*) for each experimental group/condition, given as a discrete number and unit of measurement
☒	☐	A statement on whether measurements were taken from distinct samples or whether the same sample was measured repeatedly
☒	☐	The statistical test(s) used AND whether they are one- or two-sided<br>*Only common tests should be described solely by name; describe more complex techniques in the Methods section.*
☒	☐	A description of all covariates tested
☐	☒	A description of any assumptions or corrections, such as tests of normality and adjustment for multiple comparisons
☐	☒	A full description of the statistical parameters including central tendency (e.g. means) or other basic estimates (e.g. regression coefficient) AND variation (e.g. standard deviation) or associated estimates of uncertainty (e.g. confidence intervals)
☒	☐	For null hypothesis testing, the test statistic (e.g. *F*, *t*, *r*) with confidence intervals, effect sizes, degrees of freedom and *P* value noted<br>*Give P values as exact values whenever suitable.*
☒	☐	For Bayesian analysis, information on the choice of priors and Markov chain Monte Carlo settings
☐	☒	For hierarchical and complex designs, identification of the appropriate level for tests and full reporting of outcomes
☒	☐	Estimates of effect sizes (e.g. Cohen's *d*, Pearson's *r*), indicating how they were calculated

Our web collection on statistics for biologists contains articles on many of the points above.

Software and code

Policy information about availability of computer code

Data collection	OpenFOAM v2112, StarCCM v2306, Python 3.10, Java 17.0.5, C ++ 17.
Data analysis	Python 3.08, 3.09, 3.10.

For manuscripts utilizing custom algorithms or software that are central to the research but not yet described in published literature, software must be made available to editors and reviewers. We strongly encourage code deposition in a community repository (e.g. GitHub). See the Nature Portfolio guidelines for submitting code & software for further information.

Data

Policy information about availability of data

All manuscripts must include a data availability statement. This statement should provide the following information, where applicable:

- Accession codes, unique identifiers, or web links for publicly available datasets
- A description of any restrictions on data availability
- For clinical datasets or third party data, please ensure that the statement adheres to our policy

The 3D models used for this research are available at https://doi.org/10.6084/m9.figshare.24787773; all the code used in this work to generate the 3D geometries, input files and data analysis is available on the following GitHub link https://github.gatech.edu/ibartol3/DOD-TA2-Aim2 and can be accessed by reasonable request .

Research involving human participants, their data, or biological material

Policy information about studies with human participants or human data. See also policy information about sex, gender (identity/presentation), and sexual orientation and race, ethnicity and racism.

Reporting on sex and gender	Sex information comes from the anonymized CT scans. Due to computational constraints simulations were performed only on the Male clusters but future research will expand to consider both sexes.
Reporting on race, ethnicity, or other socially relevant groupings	There was no information associated nor used regarding race, ethnicity, or other socially relevant groupings.
Population characteristics	No human participants were involved in this study, as all the CT scans come from publicly available data.
Recruitment	N/A
Ethics oversight	N/A

Note that full information on the approval of the study protocol must also be provided in the manuscript.

Field-specific reporting

Please select the one below that is the best fit for your research. If you are not sure, read the appropriate sections before making your selection.

☒ Life sciences ☐ Behavioural & social sciences ☐ Ecological, evolutionary & environmental sciences

For a reference copy of the document with all sections, see nature.com/documents/nr-reporting-summary-flat.pdf

Life sciences study design

All studies must disclose on these points even when the disclosure is negative.

Sample size	Sample size was chosen based on the most publicly available CT scans. For choosing subgroups of the CT scans a k-means clustering algorithm was used and the number of cluster was decided to be three on each dimension based on wanting to have one group representing the mean and two other groups below and above the mean, for each dimension analyzed.
Data exclusions	As mentioned also in the manuscript, there were multiple CT scans excluded from the reconstruction analysis due to not meeting the minimum quality requirements for reconstruction.
Replication	We provide by request all the necessary code to reproduce this research. Moreover, in the manuscript we provide with great detail the reconstruction process used, and all the parameters used for the simulation. Also, one of the co-authors who was not involved in the CFD/CFPD simulation part, tested the code to see that was easy to use and perform a simulation out of this. We realized that the code only works for that version of StarCCM+ and OpenFOAM mentioned or newer, but older versions may have incompatibilities.
Randomization	The part of the research that included experimental groups, those were decided by an importance analysis using a Random Forest regressors and after that a clusterization process on the two most important variables was performed using k-means algorithm.
Blinding	All the investigators were blinded to the data and did not perform any selection of datasets based on personal criteria.

Reporting for specific materials, systems and methods

We require information from authors about some types of materials, experimental systems and methods used in many studies. Here, indicate whether each material, system or method listed is relevant to your study. If you are not sure if a list item applies to your research, read the appropriate section before selecting a response.

Materials & experimental systems

n/a	Involved in the study	
☒	☐	Antibodies
☒	☐	Eukaryotic cell lines
☒	☐	Palaeontology and archaeology
☒	☐	Animals and other organisms
☒	☐	Clinical data
☒	☐	Dual use research of concern
☒	☐	Plants

Methods

n/a	Involved in the study	
☒	☐	ChIP-seq
☒	☐	Flow cytometry
☒	☐	MRI-based neuroimaging

Plants

Seed stocks	*Report on the source of all seed stocks or other plant material used. If applicable, state the seed stock centre and catalogue number. If plant specimens were collected from the field, describe the collection location, date and sampling procedures.*
Novel plant genotypes	*Describe the methods by which all novel plant genotypes were produced. This includes those generated by transgenic approaches, gene editing, chemical/radiation-based mutagenesis and hybridization. For transgenic lines, describe the transformation method, the number of independent lines analyzed and the generation upon which experiments were performed. For gene-edited lines, describe the editor used, the endogenous sequence targeted for editing, the targeting guide RNA sequence (if applicable) and how the editor was applied.*
Authentication	*Describe any authentication procedures for each seed stock used or novel genotype generated. Describe any experiments used to assess the effect of a mutation and, where applicable, how potential secondary effects (e.g. second site T-DNA insertions, mosiacism, off-target gene editing) were examined.*
